# Supplementary material for: Splice-Junction-Based Mapping of Alternative Isoforms in the Human Proteome
Source: Cell Rep. Author manuscript; Available in PMC 2020 Jan 15. (PMC6961840; doi:10.1016/j.celrep.2019.11.026)
Supplement: 2 [file NIHMS1546469-supplement-2.zip › DF1/PXD010154/Liver/Liver_12_NUP153_RIPSIVSSPLNSVDSQYPPVQR.pdf]

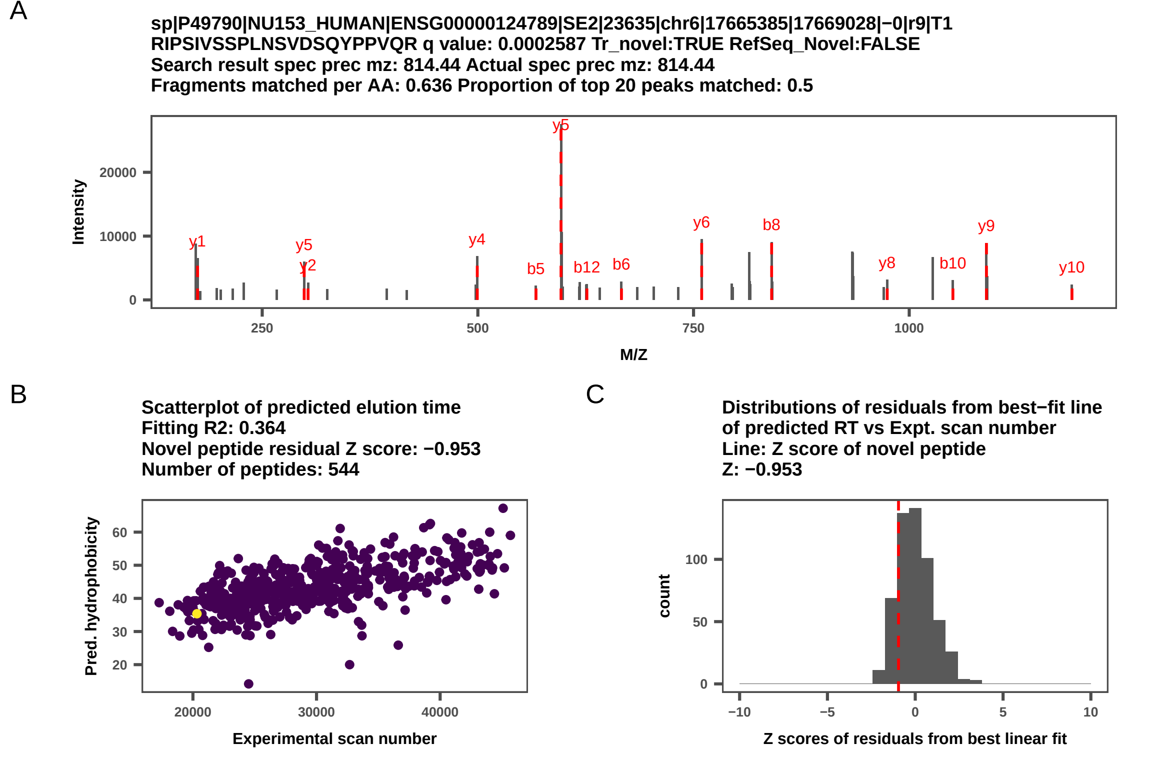
A
sp|P49790|NU153_HUMAN|ENSG00000124789|SE2|23635|chr6|17665385|17669028|−0|r9|T1
RIPSIVSSPLNSVDSQYPPVQR q value: 0.0002587 Tr_novel:TRUE RefSeq_Novel:FALSE
Search result spec prec mz: 814.44 Actual spec prec mz: 814.44
Fragments matched per AA: 0.636 Proportion of top 20 peaks matched: 0.5
Intensity
20000
10000
0
y1
y5
y2
y4
b5
y5
b12
b6
y6
b8
y8
b10
y9
y10
250
500
750
1000
M/Z
B
Scatterplot of predicted elution time
Fitting R2: 0.364
Novel peptide residual Z score: −0.953
Number of peptides: 544
Pred. hydrophobicity
60
50
40
30
20
20000
30000
40000
Experimental scan number
C
Distributions of residuals from best−fit line
of predicted RT vs Expt. scan number
Line: Z score of novel peptide
Z: −0.953
count
100
50
0
−10
−5
0
5
10
Z scores of residuals from best linear fit
